# An automated endpoint control platform for a cyclone concentration pipette

**DOI:** 10.1007/s44211-026-00967-1

**Published:** 2026-09-17

**Authors:** Ruying Wang, Chenyu Zhou, Ryoichi Ohta, Kazuma Mawatari

**Affiliations:** Waseda University - Kitakyusyu Campus, Kitakyusyu, Japan

**Keywords:** Automated control, Cyclone concentration, Microfluidic, Volume control

## Abstract

In the point-of-care, microfluidic, and biochemistry areas, the sample volume is becoming as small as µL order. In this study, we introduce a cyclone concentration pipette that integrates quantitative concentration and sample ejection functions for liquid volume conversion (mL to µL). The concentration pipette is a three-scale polypropylene (PP) tube comprising a cm-scale cyclone concentration chamber, a mm-scale channel for keeping the 10 µL liquid, and a µm-scale orifice (105 µm ± 6 µm) for ejecting liquid. The cyclone concentration system requires continuous observation to determine the endpoint (10 µL). This work introduces an automated control system designed for precise control of a 10 µL volume. The system is built on a Raspberry Pi platform with a Logitech C920 USB camera (1920 × 1080). It monitors the tube in real-time using three parallel detection methods: a convolutional neural network (CNN) for liquid state classification, a MOG2 background subtractor for droplet movement detection, and temporal pixel variance analysis across a 40-frame rolling window for flow state monitoring. When no activity is detected by any method for 30 consecutive seconds, the Raspberry Pi automatically closes a KOGANEI proportional pneumatic valve via a PWM-to-voltage converter (0–10 V). The system automatically terminated concentration without manual intervention. For the water concentration, the mean final volume was 10.6 μL with a standard deviation of 1.65 μL, corresponding to a coefficient of variation (CV) of 15.6% and a bias of + 6.0% relative to the target volume of 10 μL (*n* = 10). The optimized imaging conditions improved the stability of endpoint detection and volume control, resulting in a final volume of 10.4 μL (CV = 8.2%) with a bias of + 4.0% relative to the 10 μL target, demonstrating acceptable endpoint control accuracy. For the Sunset Yellow solution, a recovery of > 90% and a concentration factor of approximately 90-fold were achieved, indicating that the automated control strategy maintained high sample recovery while achieving efficient concentration.

**Graphical abstract:**

**Supplementary Information:**

The online version contains supplementary material available at https://doi.org/10.1007/s44211-026-00967-1.

**Figure:**
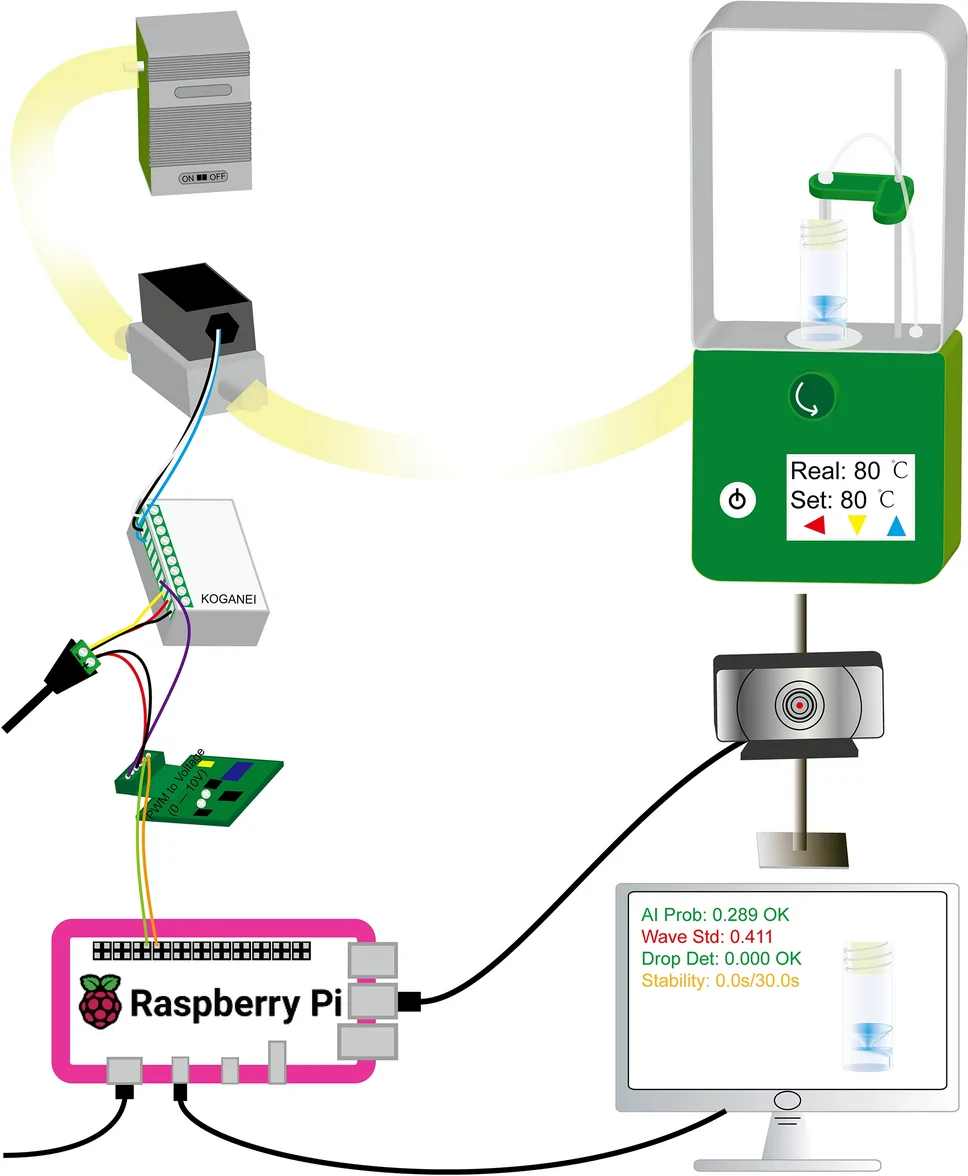

## Introduction

In analytical processes, sample concentration is an essential pretreatment step, especially if the concentration level of the target analytes is low. In many cases, environmental [1], biological [2, 3], and chemical samples [4] need to be concentrated before they are analyzed spectroscopically, chromatographically, or electrochemically in order to increase sensitivity in the detection and to decrease the effect of the matrix. Therefore, the formulation of reliable and efficient concentration methods [5] is still an important research field in analytical science.

The cyclone-based concentration is one of the concentration methods that is gaining attention due to its ability to remove solvent without membranes or external absorbents. Solvent evaporation can be accelerated under reduced pressure while nonvolatile analytes are kept in a small collection volume by generating a vortex flow. Cyclone concentration can process a wide variety of sample types, and does not suffer from fouling like membrane methods [6]. It has recently been shown to be useful for concentrating dyes, biomolecules, and trace contaminants before measurement [7].

However, the working of cyclone concentration devices is still mainly reliant on manual observation. The operator needs to be vigilant and constantly watch the liquid’s behavior during concentration and decide when to stop the process. Too short a termination time will cause inadequate enrichment, while too long a time can cause the sample to dry out, some of the analyte to be lost, or a lower recovery of the analyte. As the volume of the sample decreases to the microliter range, the movement of the liquid becomes more subtle, and endpoint detection is dependent on the operator’s experience and can vary from experiment to experiment.

This work introduces an automated concentration device comprising a three-scale polypropylene (PP) concentration pipette and a vision-based automated control system. The concentration pipette employs a cyclone method to concentrate samples from mL to µL without the need for a membrane and while maintaining sample temperature [8, 9]. This increases sample concentration, thereby improving detection sensitivity and accuracy, and reducing sample waste. Furthermore, the concentration pipette combines three scales: cm, mm, and µm, concentrating the sample from the centimeter-scale cyclone concentration chamber to the millimeter-scale collection channel. This microchannel has a volume of 10 µL. Due to Laplace pressure and the pinning effect at the micrometer-scale orifice [10], the 10 µL of liquid can be kept within the microchannel. Because the average concentration rate of the liquid in the microchannel is 20 times slower than in the cm chamber, and because the microchannel is located near the edge of the concentration pipette, the cyclone stops at the microchannel, making observation with a camera very convenient [11]. When the cap is closed, the applied pressure exceeds the Laplace pressure, allowing the 10 µL droplet to be ejected from the orifice at the bottom.

We developed an automatic control system for this concentration pipette, designed to automatically monitor whether the volume has reached 10 µL and automatically shut off the pneumatic pump valve. This system decreases the labor costs of continuous manual monitoring and the errors introduced by manual monitoring (inconsistencies in operator judgment often lead to inconsistent endpoints, affecting recovery rates and repeatability). The control system uses three parallel computer vision methods and image analysis to detect whether the liquid sample has been concentrated to 10 µL. The system combines a Raspberry Pi, a USB camera, and a proportional valve to automatically shut off the pneumatic pump valve when 10 µL is reached. This automatic control system is easy to operate, inexpensive, reduces labor costs, and improves the repeatability of the final volume.

## Experimental methods

### Design and fabrication of the concentration pipette and the control system

Our research group independently developed the concentrated pipette used in this study. Before loading, the outlet was sealed with four layers of parafilm to prevent leakage during cyclone concentration. The cyclone concentration process takes place in a centimeter-scale cylindrical chamber. Before the operation, the cap of the concentrated pipette is opened, and a screw plug with a hollow channel is inserted. This hollow channel is connected to an air pump, which continuously draws air from the pipette, creating a negative pressure inside the concentration device. External air then enters the pipette through the hollow channel, generating airflow and inducing cyclone motion in the liquid.

**Fig. 1:**
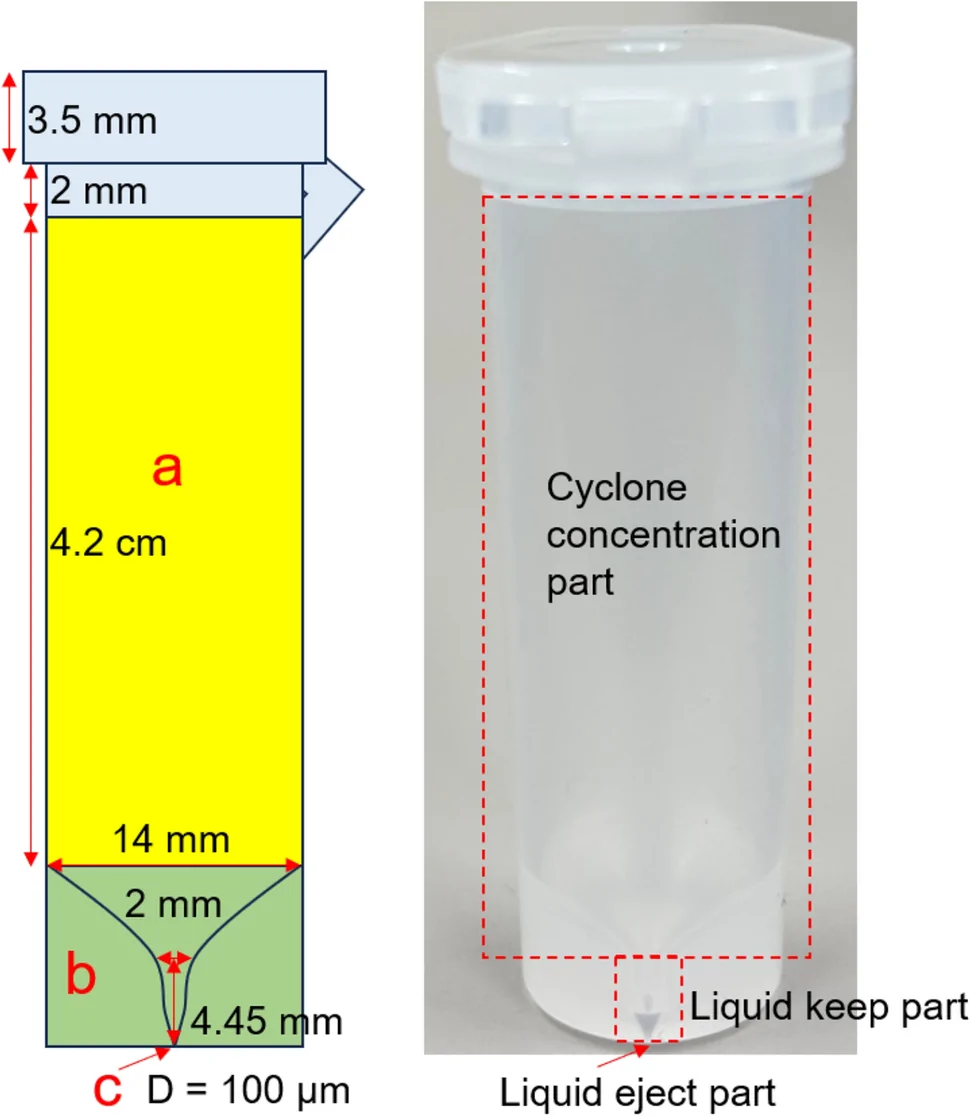
The design of the concentration pipette

The cyclone motion promotes solvent evaporation, gradually reducing the liquid volume and thus increasing the analyte concentration. When 1 mL of the sample is concentrated to 10 μL, the theoretical concentration factor is 100-fold. During the concentration process, the liquid first forms a cyclone within the cylindrical chamber. As the volume decreases, the remaining liquid enters a funnel-shaped area at the bottom of the chamber, where it continues to cyclone until the liquid completely enters the microchannel. The concentration device consists of a cylindrical (yellow part, a), centimeter-sized chamber (4.2 cm high, 1.4 cm in diameter), a transparent, millimeter-sized channel (green part, b, 10 μL volume), and a 100 μm outlet at the bottom (c) (Fig. 1). The centimeter-sized chamber provides a site for cyclone concentration. The off-axis channel design facilitates direct observation and volume measurement while stopping the cyclone, storing the 10 μL of liquid within the channel.

The microfluidic channel almost stops the cyclone. In a microchannel with a top diameter of 2.0 mm and a bottom diameter of 100 μm, conventional cyclonic concentration cannot occur, primarily because the millimeter-scale channel is located at the edge rather than in the center, thereby preventing vortex formation. Due to the small size and low Reynolds number, the flow is typically laminar, making it easier to observe the air–liquid interface [12]. When the cap is closed, the applied pressure overcomes the Laplace pressure and pinning effect at the bottom outlet, allowing the 10 μL of concentrated liquid to be completely injected (Fig. S1). The device is constructed from polypropylene (PP) to minimize sample adsorption. It is manufactured using a precision injection molding process, where molten PP is injected into a micro-machined mold and cooled to form the final shape. This molding process achieves dimensional accuracy of ± 2–3 μm and a mold surface roughness of Ra 0.01–0.03 μm.

The volume of the microchannel is 10 μL. Because the narrow structure of the microchannel prevents the formation of a cyclone, the liquid tends to remain still after entering the microchannel. Therefore, when no further liquid flow was observed within the microchannel, the sample volume had reached 10 μL.

After concentration, the concentrated sample was collected by opening the 100 μm outlet at the bottom of the pipette. After removing the parafilm, the cap was quickly closed to create internal pressure. This pressure overcame the Laplace pressure and pinning effect at the outlet, allowing the 10 μL of concentrated sample within the microchannel to be completely expelled.

During manual operation, frequent pauses are required to repeatedly visually inspect the tiny droplets forming in the inverted conical chamber. Furthermore, the concentrator pipette must be removed from the spiral plug to allow the droplets to coalesce within the chamber, enabling the operator to visually estimate the droplet volume. Since the droplet volume at this point is approximately 5 μL (about 50% of the target volume), careful monitoring is essential. Therefore, endpoint determination is labor-intensive, subjective, and highly dependent on operator experience, especially during prolonged concentration processes.

In previous cyclone concentration systems, the endpoint was manually determined by observing the cessation of liquid movement. In this study, this manual operation has been replaced by an automated control system that uses three independent image analysis methods to assess liquid activity. The vacuum pump automatically stops only when all three methods meet the endpoint criteria.

As for recovery and concentration factor, there is no huge difference between manual control and automated control. As for operation time and operator dependence, the automated control is better than manual control. This method eliminates the need for continuous operator supervision, improves the objectivity of endpoint determination, reduces manual operation, and lays the foundation for future fully automated sample pretreatment systems.

An automated control system was used to monitor the concentration process, which consisted of the Logitech C920 camera, the Raspberry Pi 5, and so on (Table 1). Figure S2 illustrates the workflow of the control system.

**Table 1:** Bill of materials used for the automated control system

Item	Component	Model	Amount	Function
1	Single-board computer	Raspberry Pi 5	1	System control and image processing
2	USB camera	Logitech C920	1	Real-time image acquisition
3	Proportional valve	KOGANEI KFPV050-2-40-FM-S4-02	1	Vacuum regulation
4	PWM-to-voltage converter	PWM-to-Voltage Module (0–10 V)	1	Converts PWM signals into an analog control voltage
5	Vacuum pump	CEP-80-GR	1	Generates negative pressure for concentration
6	Concentration device	Concentration pipette	1	Sample concentration
7	Display monitor	LCD monitor	1	Real-time monitoring and visualization
8	Power supply	12 V DC power supply	1	Power source for the valve and peripherals
9	Tubing	PTFE/Silicone tubing	1 set	Pneumatic connection
10	USB cable	USB 2.0/3.0 cable	1	Camera connection
11	Concentration system	CEV1B-GR	1	Generate Cyclone and heating

**Fig. 2:**
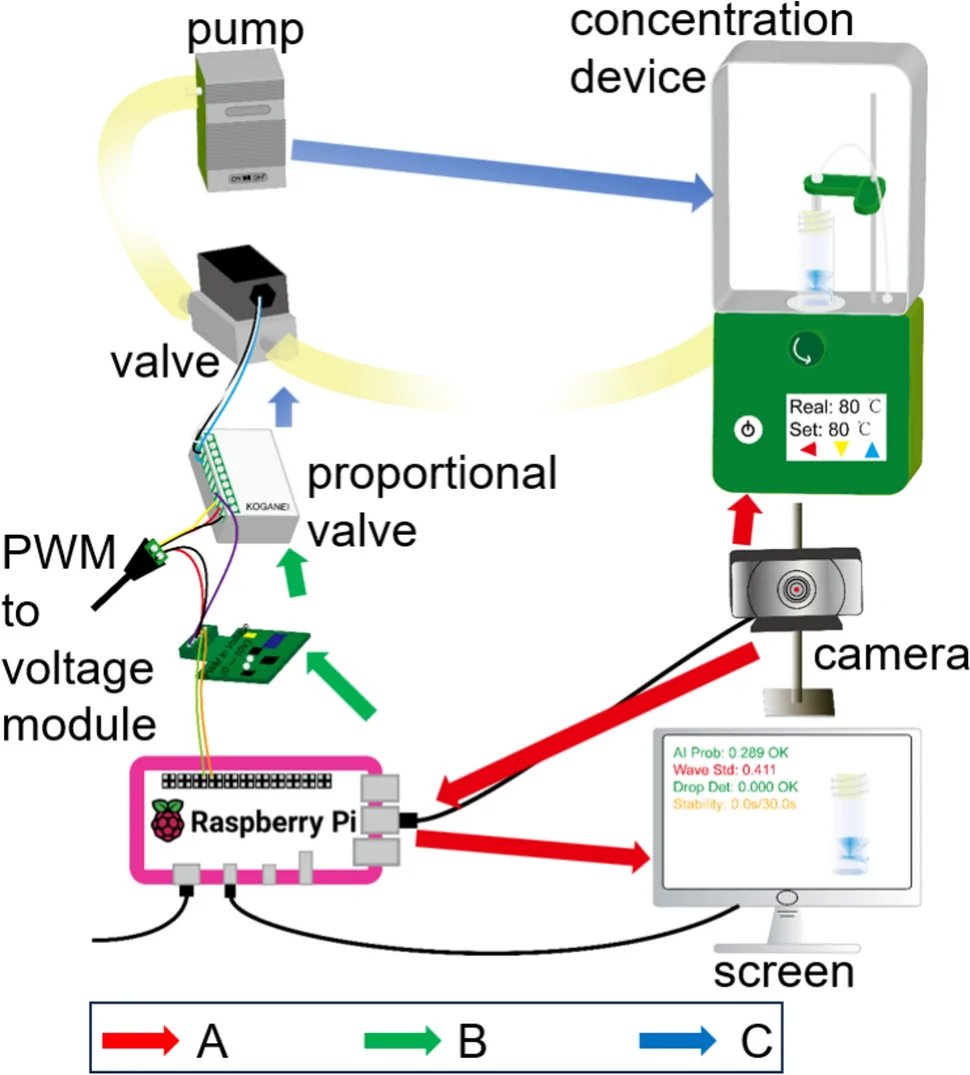
The signal logic of the whole system

As shown in Fig. 2, there are five parts of the automated control system: a Raspberry Pi 5 single-board computer; a Logitech C920 USB camera (1920 × 1080) for real-time image acquisition; a commercialized evaporation system (CEV1B-GR, BioChromato, Inc) and a vacuum pump (CEP-80-GR); a KOGANEI proportional pneumatic valve with PWM-to-voltage converter (0–10 V); and an LCD monitor.

The camera takes a video of the cyclone concentration and sends frames to the Raspberry Pi. Live data is displayed on the monitor, which is connected to the Raspberry Pi (red arrow, A). When the volume of the sample reaches 10 µL, the Raspberry Pi outputs a PWM signal converted to a 0–10 V analogue voltage by a PWM-to-voltage module to drive the KOGANEI proportional valve (green arrow, B). When the stop condition is met (0 V output), the pump ceases, and the cyclone concentration process is complete (blue arrow, C).

### Hardware assembly

**Fig. 3:**
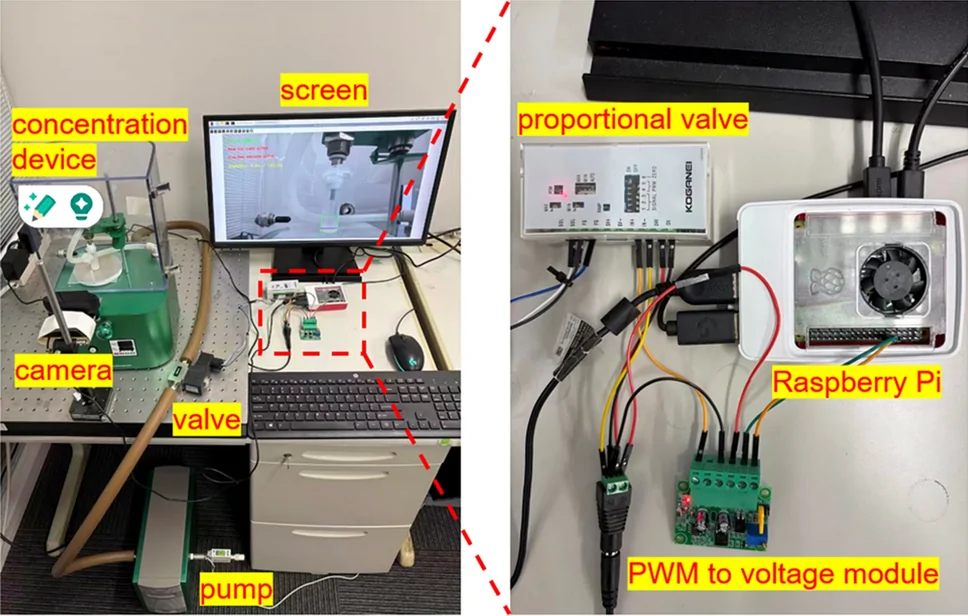
Hardware connection

As shown in Fig. 3, connect the air pump to the KOGANEI proportional valve using silicone tubing. Connect the KOGANEI proportional valve outlet to the concentration system using another silicone tube. Insert a silicone tube into a spiral plug that attaches to the concentration pipette. Mount the Logitech C920 camera on the adjustable stand, directly in front of the concentration system. Adjust the camera position so that the concentration pipette is centered in the field of view. Connect the proportional valve’s control input to the output of the PWM to voltage converter (0-10 V). Connect the converter’s signal input to GPIO 18 of the Raspberry Pi 5, ensuring that the converter and Raspberry Pi share a common ground. Connect the Logitech C920 camera to the Raspberry Pi 5’s USB 3.0 port. Connect the Raspberry Pi to a monitor and power supply (Fig. S4).

#### Pump control logic

The camera captures images of cyclone concentration and transmits them to a display via a Raspberry Pi, allowing the display to show the cyclone concentration process in real time. A timestamp records the most recent frame triggered by any detection method. Each frame is evaluated for the elapsed quiet time. When the three judgment parameters remain silent for ≥ 30 s, the Raspberry Pi PWM duty cycle is set to 0.0, and the signal is transmitted to the proportional valve via the PWM to voltage module. The valve closes, and the cyclone concentration process terminates.

#### Pneumatic control

The Raspberry Pi controls the KOGANEI proportional valve via pin GPIO 18, outputting a 2000 Hz PWM signal. A PWM-to-voltage converter module translates this signal to a 0–10 V analogue output that drives the valve. A duty cycle of 1.0 fully opens the valve (maximum pressure); 0.0 closes it completely (zero pressure). The pump applies negative pressure to the concentration chamber during the concentration process. When the vision system confirms dispensing completion, the GPIO output is set to 0.0, closing the valve and stopping the pump.

### Control method

#### CNN-based liquid cyclone on–off classification

A CNN with a spatial attention mechanism was used to classify each frame as liquid present or liquid absent. Compared with.

conventional image-processing methods, the CNN automatically extracts hierarchical image features and enables robust recognition of liquid patterns under varying illumination and background conditions. The spatial attention module enhances informative liquid-related regions while suppressing irrelevant background features. The refined feature map is obtained by

1 $$F{\prime} = A \odot F$$

where F and F′ denote the original and refined feature maps, respectively, A is the spatial attention map, and ⊙ represents element-wise multiplication.

During inference, the ROI is smoothed using a Gaussian filter, converted from the BGR color space to the RGB color space, resized to 316 × 117 pixels, and directly input into the trained CNN model. To accelerate inference on the Raspberry Pi, mixed float16 precision is used. The CNN outputs the probability of liquid presence. During system optimization, a decision threshold of 0.45 is chosen to distinguish between the presence and absence of liquid.

#### Background subtraction for droplet detection

A Gaussian Mixture Model (MOG2)-based background subtraction algorithm was employed to detect moving droplets. MOG2 models the background rather than the droplets themselves. The intensity of each pixel is represented by a mixture of Gaussian distributions, allowing pixels with stable intensities to be continuously incorporated into the background model, whereas transient intensity changes caused by moving droplets are segmented as foreground. Consequently, droplet detection is transformed into the problem of detecting deviations from the estimated background rather than directly identifying droplet features. Therefore, droplet motion can be detected by identifying foreground pixels without requiring a predefined intensity threshold.

The background model is continuously updated according to the following formula:

2 $${B}_{t}= (1 - \alpha ) {B}_{t-1}+ \alpha {I}_{t}$$

where ${B}_{t}$ is the estimated background model for frame *t*, ${B}_{t-1}$ is the background model for the previous frame, ${I}_{t}$ is the current image, and α is the learning rate used to control the adaptive speed of the background model.

In short, there are 6 steps: Build the background model; Update the background continuously; Compare each frame with the background; Extract foreground pixels; Count foreground pixels; Determine droplet passage.

The ROI was defined based on both the geometry of the concentration pipette and the observed liquid behavior during concentration. Specifically, the ROI covers the lower part of the concentration chamber, the funnel-shaped slope along which droplets rotate, and the 10-μL microchannel at the bottom of the pipette. Because the volume of the microchannel is predefined as 10 μL by the device design, the position of the liquid level corresponding to the target final volume of 10 μL is known from the device geometry. We marked this position with a blue reference line within the ROI. The blue line divides the ROI into an upper region and a lower region. The upper region accounts for 68% of the ROI and contains the cyclone chamber and funnel-shaped slope, where droplets continue to move during concentration. The lower region corresponds to the 10-μL microchannel, where no cyclone is present, and the liquid is stationary at the target endpoint. Therefore, MOG2-based droplet detection was applied only to the upper 68% of the ROI, where droplet motion provides information about whether concentration is still proceeding. This definition ensures that the MOG2 analysis focuses on the dynamically active region above the predetermined 10-μL endpoint while excluding the stationary final-volume microchannel.

#### Temporal pixel variance for flow detection

Temporal pixel variance analysis was performed to detect sustained liquid motion that may not produce distinct droplets. A rolling buffer containing the most recent 40 grayscale frames was maintained. The temporal standard deviation of each pixel was calculated, and the average standard deviation over the entire ROI was used as a flow index:

3 $$ Flow\,Index = (1/HW)\Sigma \sigma (x,y) $$

where σ(x,y) is the temporal standard deviation of pixel intensity at position (x,y) over the image sequence, and H and W are the height and width of the ROI, respectively.

When liquid is flowing, pixel intensities continuously change over time, resulting in a larger temporal standard deviation. In contrast, stationary regions exhibit nearly constant pixel intensities and therefore produce a low temporal standard deviation. Because the flow index integrates temporal image variations over approximately 1–2 s of video, it is insensitive to isolated frame disturbances while remaining sensitive to sustained liquid motion. A flow index threshold of 0.48 was empirically selected as a balance between suppressing background fluctuations and maintaining sufficient sensitivity for detecting weak continuous liquid flow. Values exceeding 0.48 were therefore regarded as indicating continuous liquid motion. A flow index threshold of 0.48 was empirically selected during preliminary experiments, as lower thresholds frequently responded to background fluctuations, whereas higher thresholds reduced the sensitivity to weak continuous liquid flow. In short, there are 4 steps to stop the cyclone flow: Store 40 consecutive grayscale frames; Calculate the temporal standard deviation of each pixel; Average all pixel deviations to obtain the flow index; Determine continuous liquid flow using the threshold (0.48).

The stop criterion was defined as 30 consecutive seconds without detectable liquid activity from any of the three monitoring methods. During preliminary experiments, we observed that weak residual liquid oscillations occasionally disappeared temporarily before reappearing. If the confirmation period was too short, these transient pauses could lead to premature pump termination. Conversely, excessively long confirmation periods unnecessarily increased the overall concentration time without improving endpoint accuracy.

Therefore, a confirmation period of 30 s was selected empirically as a practical compromise between robustness and operational efficiency. This criterion effectively suppressed false endpoint detection while maintaining reliable automatic operation.

### Combined three methods

None of these three image analysis methods is designed to directly measure whether the liquid volume has precisely reached 10 μL. Instead, each method provides information about specific aspects of the concentration process.

First, during concentration, residual droplets can become very small or remain on the funnel-shaped slope, or sometimes rotate to the side of the cyclone chamber farther from the camera. In these situations, the local liquid signal may temporarily become weak even though droplets are still present and moving. Without considering droplet motion, this state could be misclassified as “no liquid activity,” leading to an erroneous endpoint determination and premature pump termination. MOG2 background subtraction was therefore used to identify the continued motion of these residual droplets and prevent this type of false endpoint determination.

Second, above the 10-μL endpoint, a different situation occurs: distinct droplet motion can disappear while subtle fluctuations of the remaining liquid surface still persist. If the disappearance of droplet motion were misclassified as the endpoint, the system would erroneously determine that the target volume had been reached and prematurely terminate the pump. Temporal pixel variance was therefore used to detect these residual liquid-surface fluctuations and prevent this second type of erroneous endpoint determination.

Third, the CNN provides complementary appearance-based information by learning general visual features, such as the overall shape and appearance of the liquid. In the present study, the CNN showed the ability to distinguish the 10 μL and >10 μL states, and was used as a complementary method together with MOG2-based droplet-motion detection and temporal pixel variance analysis. The current CNN dataset did not specifically include a large number of wall-adhered droplets, bubbles, or turbid samples; incorporating such images into the training dataset in future work could further improve its robustness to complex visual interference.

Thus, the three methods were designed to provide complementary information rather than to perform the same type of detection: the CNN evaluates overall appearance, MOG2 detects droplet motion, and temporal pixel variance detects residual temporal fluctuations. Their combination provides multiple visual criteria for endpoint determination rather than relying on a single image feature.

### Sunset yellow concentration experiment

A 40 μM Sunset Yellow aqueous solution was prepared. 1 mL of the sample was added to the concentration pipette and concentrated to approximately 10 μL. The concentrated sample was then completely ejected into a quartz cuvette and diluted to 2 mL with pure water. The absorbance was measured using a UV–Vis spectrophotometer (Fig. 5a). The concentration experiments were repeated 3 times. A characteristic absorption peak of Sunset Yellow was observed at 482 nm. As a control, 1 mL of 40 μM Sunset Yellow standard solution was diluted to 2 mL and measured under the same conditions.

## Results and discussion

### The performance of the CNN model

The CNN was trained offline using images extracted from concentration experiments and manually labeled according to the liquid-volume state. Two classes were defined: “10 μL”, corresponding to images in which the concentrated liquid had reached the designated 10 μL endpoint position, and “>10 μL”, corresponding to images in which the liquid was still above the designated 10 μL position. The 10 μL endpoint images consisted of 14 independently acquired endpoint images. For the >10 μL class, frames were extracted from three independent concentration videos. From each video, 60 representative frames were sampled, resulting in 180 images for the >10 μL class.

To reduce the possibility of information leakage among the 10 μL images, the 14 independent endpoint images were first divided at the original-image level into training, validation, and test sets at a ratio of approximately 9:2:3. Image augmentation was then applied only within the corresponding subset. Thus, the augmented images generated from a given original endpoint image were not distributed across different subsets. The numbers shown in the table represent the final numbers of images after augmentation. The final dataset contained 224 training images, 50 validation images, and 60 test images. The detailed composition was as shown in Table S1.

The trained model was evaluated on the test dataset. The 10 μL class was defined as the positive class for the calculation of precision, recall, and F1-score. The resulting test-set performance was shown in Table S2, the accuracy is 81.67%, precision is 100%, recall is 66.67%, and F1-score is 80.00%.

The corresponding confusion matrix was shown in Table S3, the CNN correctly identified 22 of the 33 actual 10 μL test images, while 11 were conservatively classified as >10 μL. Importantly, none of the 27 >10 μL test images was incorrectly classified as 10 μL. Thus, in the test dataset, the CNN did not produce a false-positive 10 μL classification, although it missed some true 10 μL states.

The relatively limited recognition of the 10 μL state is attributable, at least in part, to the limited number of images representing the 10 μL endpoint state. Because the visual characteristics of the 10 μL state can vary depending on the liquid distribution and imaging conditions, a larger and more diverse set of training images is expected to improve the model’s ability to generalize to this state. As additional experimental data are accumulated, the training dataset can be expanded, and the CNN can be optimized to improve endpoint recognition performance.

The CNN was used to provide appearance-based information on whether the liquid state had reached the designated 10 μL endpoint, rather than to quantitatively determine the final volume. Therefore, the CNN was used as one complementary appearance-based method in the endpoint-control framework, rather than as a standalone volume classifier. In the present study, visually complex conditions such as wall-adhered residual liquid, bubbles, and turbid samples were not sufficiently represented in the training dataset. Incorporating such images in future work could further improve the CNN’s ability to recognize different visual states encountered during concentration and broaden its applicability to more diverse sample conditions.

### Effectiveness of combining the three image-analysis methods

Table 2 shows that the CNN-only approach was not as effective as the combined three methods. Among five concentration experiments, successful automatic pump stop was achieved in only three cases (60%), resulting in a mean final volume of 15.0 μL and a CV of 33.3%. In contrast, the combined three-methods framework successfully terminated all ten experiments (100%), with a mean final volume of 10.6 μL and a CV of 15.6%. Successful automatic pump stop was defined as a concentration run in which the control system successfully detected the endpoint condition and issued a pump-stop command, regardless of the final measured volume. These results suggest that the combination of three methods provides more reliable endpoint control than the CNN-only approach.

**Table 2:** Comparison between the individual CNN method and the combined three methods

Parameter	CNN only	Combined three-methods
Number of experiments	5	10
Successful automatic pump stop	3/5 (60%)	10/10 (100%)
Automatic pump stop failure	2/5 (40%)	0/10 (0%)
Mean final volume of successful runs (μL)	15.0	10.6
CV of successful runs	33.3%	15.6%

### Automatic endpoint determination

The automated control system was specifically developed for the cyclone concentration pipette, and therefore, its design was closely tailored to the characteristics of the concentration process. During cyclone concentration, as the liquid level approaches the microchannel, the remaining liquid continuously rotates within the inverted conical chamber (Video S1). At this stage, the residual droplet becomes extremely small and appears intermittently. Therefore, reliable endpoint determination requires continuous detection of the appearance and disappearance of these tiny droplets. Furthermore, when only a small amount of liquid remains above the microchannel, the liquid still exhibits very weak motion. To ensure that the concentration process continues until the target volume of 10 μL is reached, the system must be able to distinguish subtle liquid motion from a completely stationary state. For these reasons, three complementary image-analysis methods were combined to provide robust endpoint determination throughout the entire concentration process.

**Fig. 4:**
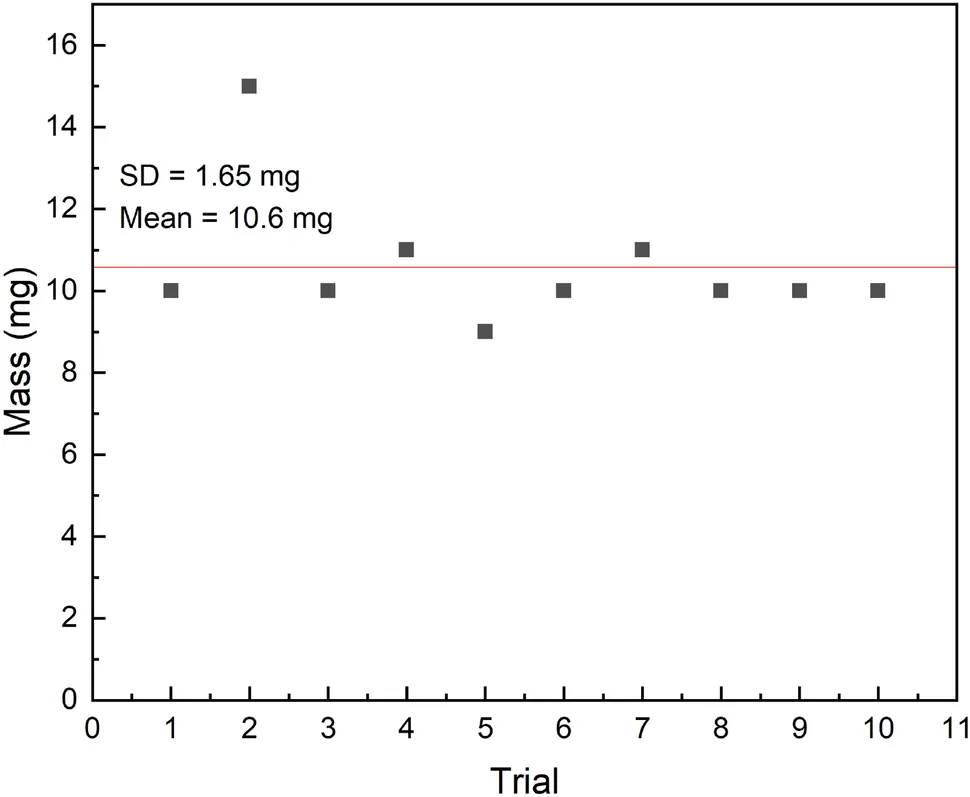
Concentrate the sample from 1 mL to 10 μL, and measure the mass of the concentrated sample (*n* = 10)

To evaluate the performance of the automated control system, ten independent concentration experiments were conducted. In each experiment, the endpoint was determined automatically by the vision-based control algorithm, which subsequently closed the proportional valve. The gravimetric measurements provide an independent verification of the approximate final volume rather than a high-precision determination of endpoint-control accuracy. The masses of the concentrated samples were measured using an electronic balance with a readability of 1 mg, resulting in values of 10, 15, 10, 11, 9, 10, 11, 10, 10, and 10 mg, corresponding to final volumes of 10, 15, 10, 11, 9, 10, 11, 10, 10, and 10 μL, respectively. The mean final volume was 10.6 μL with a standard deviation of 1.65 μL, corresponding to a coefficient of variation (CV) of 15.6% and a bias of + 6.0% relative to the target volume of 10 μL (Fig. 4).

These results demonstrate that the proposed control system was capable of terminating the concentration process at a final volume close to the target value of 10 μL (Fig. S3). One experiment terminated at approximately 15 μL, which may have resulted from external disturbances affecting image acquisition, such as changes in ambient illumination caused by nearby human activity.

To minimize the influence of environmental light fluctuations and further improve system robustness, regarding the camera, the Logitech C920 camera was set to the same imaging conditions using the Linux V4L2 (v4l2-ctl) interface. Since the relative position between the camera and the concentration pipette remained constant throughout all experiments, autofocus was disabled, and the focus_absolute parameter was fixed at 120 to obtain a clear image of the concentration pipette. The exposure_absolute parameter was manually adjusted and fixed at 70. Furthermore, the contrast and sharpness were set to 100 and 255, respectively.

Furthermore, the entire imaging system was housed in a dark box (simple desktop dark box, BBX-042-8070-04, 900 × 750 × 900 mm), equipped with a dedicated LED light source to provide constant illumination throughout all experiments. These fixed imaging conditions provided stable and repeatable images, thereby improving the reliability of subsequent three-method image analysis.

After the above optimization, the concentration chamber and microchannel became clearer in the captured images. The optimized system was further evaluated by performing three independent concentration experiments using diluted water. The mass of each collected concentrated sample was measured using an electronic balance (ASR224/E, minimum display increment: 0.0001 g, corresponding to 0.1 mg), resulting in values of 9.5, 10.5, and 11.2 mg. The mean final volume was 10.4 μL, with a coefficient of variation (CV) of 8.2% and a bias of + 4.0% relative to the target volume of 10 μL. These results demonstrate that the optimized imaging and control system achieved improved stability for endpoint detection and volume control.

### Sunset Yellow was used for validation

**Fig. 5:**
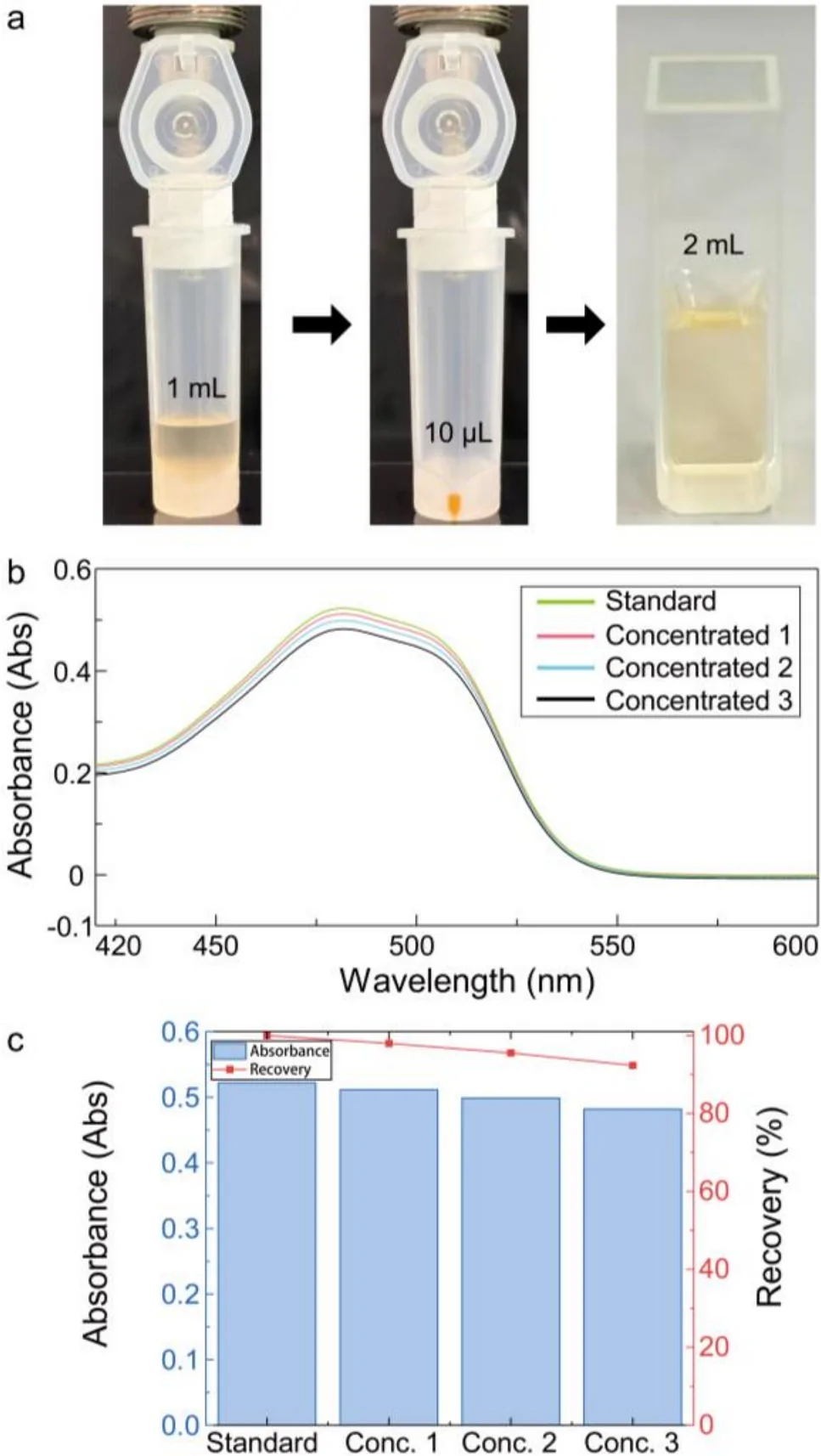
The recovery of the concentration pipette. **a** concentrate 1 mL of Sunset Yellow solution to 10 μL, then dilute to 2 mL for measurement. **b** the UV-spectra of the Standard solution and four concentrated samples. **c** the recovery is calculated based on the absorbance

Sunset Yellow was used as the model analyte for the analytical performance of the automated concentration system. The 1 mL sample solution was automatically concentrated to about 10 μL (corresponding to a theoretical concentration factor of 100) and then diluted 20-fold to 2 mL, finally analyzed using UV–Vis spectrophotometry. As shown in Fig. 5b, the absorbance of the concentrated and diluted samples was near that of the standard solution, indicating the concentration process has enriched the analyte successfully. According to Beer-Lambert Law, the recovery of the concentration pipette can be calculated as:

4 $$ {\mathrm{Recovery}} = \frac{{A_{{\mathrm{f}}} }}{{A_{i} }} \times 100\% $$

(*A*_f_ is the absorbance of the concentrated sample: 0.51, 0.49, 0.48, *A*_i_ is the absorbance of the standard solution: 0.52), which was higher than 90%, and the concentration factor was nearly 90-fold (Fig. 5c). The high recovery indicates that the automatic termination was close to the desired endpoint.

We investigated whether colored, transparent, and turbid samples affected the performance of the automated control system. This study tested colored (Sunset Yellow) and transparent (ultrapure water) samples. Experiments showed that this system can be applied to colorful samples. Also, stable illumination and optimized camera settings ensured reliable endpoint detection for transparent samples. However, untreated river water contains salt and impurities, which may adsorb onto the inner wall of the concentration pipette during the concentration process (Fig. S5). These residues can affect image analysis and reduce the accuracy of endpoint determination. Therefore, direct automated endpoint control is currently not suitable for untreated river water samples. However, with appropriate sample pretreatment, such as filtration to remove suspended impurities or other clarification steps to reduce crystal formation, this automated control system holds promise for application to environmental water samples. This study did not include samples that generated bubbles, which remains a direction for future optimization.

### Comparison between manual and automated control

As shown in Table 3, the values for manual operation were obtained from previous manual concentration experiments and represent typical operating conditions. The automated-operation values were obtained using the automated control system described in this study.

**Table 3:** Comparison between manual and automated control

Parameter	Manual operation	Automated operation
Total operation time	~150 min	~120 min
Operator attention time	~30 min	0 min
Manual interventions	1 – 3 times	0
Endpoint termination	Manual	Automatic
Final-volume reproducibility (CV)	1.8%	8.2%
Recovery	> 90%	> 90%
Concentration factor	> 90-fold	~90-fold

Manual operation refers to the conventional procedure in which the operator visually monitors the liquid state and manually determines whether the target final volume has been reached. Before the concentration experiment, a reference mark corresponding to the target endpoint is indicated on the device. During concentration, we periodically observe the liquid distribution and visually judge the remaining liquid volume relative to this reference position. The operator manually pauses the concentration process when necessary, because residual droplets can remain on the funnel-shaped slope or in other parts of the concentration chamber, and collects the residual droplets toward the final collection region before reassessing the overall liquid volume. This observation, judgment, and manual droplet-collection procedure may need to be repeated during a single concentration experiment.

In contrast, in the automated operation, the endpoint is determined by the vision-based control system, and the proportional valve is automatically closed when the endpoint condition is satisfied, without manual observation or manual droplet collection.

These results demonstrate that the practical advantage of the proposed system is the substantial reduction in operator involvement while maintaining recovery and concentration factor at comparable levels to manual operation. The total operation time was reduced from approximately 150 to 120 min, corresponding to an approximately 20% reduction. Approximately 30 min of operator attention was required during manual operation, whereas the automated system required no continuous operator attention. Manual interventions, including pausing the process, visually assessing the liquid volume, and manually collecting residual droplets, were also eliminated. Thus, the approximately 120-min automated concentration process could proceed without continuous operator supervision, while recovery and concentration factor remained comparable to those obtained with manual operation (> 90% and approximately 90-fold, respectively).

The final-volume reproducibility (CV) of the final volume was 1.8% for manual operation and 8.2% for automated operation. Therefore, manual operation showed better final-volume reproducibility.

This difference is mainly related to the different endpoint-control mechanisms used in the two modes. During manual operation, the operator continuously observes the liquid level and can intervene and adjust the concentration process when the liquid approaches the 10 μL target, allowing the endpoint to be manually corrected. In contrast, the automated system determines the endpoint from image-based detection and automatically stops the concentration process once the predefined endpoint condition is satisfied. The current image-based endpoint detection and control strategy therefore does not provide the same iterative fine adjustment that is possible during manual operation, resulting in a larger variation in the measured final volume.

## Conclusion

An automated control system for precise 10 µL volume control has been presented. The system integrates a concentration pipette with a Raspberry Pi-based control platform, combining CNN-based liquid classification, MOG2 background subtraction, and temporal pixel variance analysis in a three-method visual detection framework. If the three methods stay silent for over 30 s, the proportional valve is closed via the GPIO PWM output. The control system can automatically stop the concentration process when the volume of the liquid reaches 10 uL. Experimental validation demonstrated a mean recovery rate of 90% and a concentration factor of approximately 90-fold of the concentration pipette. This control system has potential applications in sample pretreatment for chemical analysis, environmental monitoring, and so on. It can reduce operator dependence.

## Supplementary Information

Below is the link to the electronic supplementary material.

Supplementary Material 1

Supplementary Material 2

## Data Availability

The source data for all figures are available upon request from the corresponding author K. Mawatari.
