# Supplementary material for: An automated endpoint control platform for a cyclone concentration pipette
Source: Anal Sci. 2026 Sep 17;42(11):1155–65. doi: 10.1007/s44211-026-00967-1 (PMC13690928; doi:10.1007/s44211-026-00967-1)
Supplement: Supplementary file 1 [file 44211_2026_967_MOESM1_ESM.docx]

**Supporting Information**

An Automated Endpoint Control Platform for A Cyclone Concentration Pipette

Ruying Wang, Chenyu Zhou, Ryoichi Ohta, and Kazuma Mawatari*

* Graduate School of Information, Production and Systems, Waseda University, Kitakyushu, Japan.

**Table of Contents**

1. Materials and reagents ……………………………………………………………………….S2
2. Apparatus …………………………………………………………………………………….S2
3. Figure S1. Concentration and ejection principle of the concentration pipette………………..S3
4. Figure S2. Software workflow……………….…………………………………………........ S4
5. Figure S3. The 10 μL precision control by the Raspberry Pi platform…………….………....S5
6. Figure S4. The connection of the control system……………………………………………..S6
7. Figure S5. Untreated river water sample adsorbs onto the inner wall of the concentration pipette…………………………………………………………………………………………S7
8. Table S1 The composition of the dataset.…………………………………………………….S8
9. Table S2 Performance on the test set…………………………………………………………S8
10. Table S3 The corresponding confusion matrix of the test set………………………………...S8

**Experimental Section**

1. **Materials and Reagents**

Sunset Yellow solution was provided by Tokyo Chemical Industry Co., Ltd. (Tokyo, Japan). Raspberry Pi 5 was purchased from Raspberry Pi Ltd. (Cambridge, UK). Logitech C920 was obtained from Logitech International S.A. (Lausanne, Switzerland). KOGANEI KFPV050-2-40-FM-S4-02 was supplied by KOGANEI Corp. (Tokyo, Japan). The PWM-to-Voltage Module (0–10 V output, commercially available).

2. **Apparatus**

The instrument used to measure the weight of a 10-mL droplet pumped out by the concentration pipette was the ASPRO Electronic Balance (Min Representation (g): 0.001). For the optimization experiments, the mass of the collected concentrated sample was measured using an electronic balance (ASR224/E, minimum display increment: 0.0001 g, corresponding to 0.1 mg). All UV spectra of Sunset Yellow solutions in this work were characterized using the V-730 UV-visible spectrophotometer from JASCO with silicon photodiode detectors, a wide wavelength range – 190 to 1,100 nm, fixed bandpass 1.0 nm for high resolution measurement, and high-speed scanning up to 8,000 nm/min.

**Supplementary Figures**

Fig. S1 Concentration and ejection principle of the concentration pipette. Liquid is kept in the microchannel due to the Laplace pressure. When the cover is closed, the pressure resists the Laplace pressure, and the liquid is ejected.

Fig. S2 Software workflow.

Fig. S3 The 10 μL precision control by the Raspberry Pi platform. Concentrate 1 mL of liquid into 10 μL. The auto control system decides the endpoint and stops the concentration process automatically.

Connect to charger

Connect to charger and monitor

Connect to

proportional valve

Fig. S4 The connection of the control system.

Fig.S5 Untreated river water sample adsorbs onto the inner wall of the concentration pipette.

| Dataset | 10 μL images | >10 μL images | Total |
| --- | --- | --- | --- |
| Training | 99 | 125 | 224 |
| Validation | 22 | 28 | 50 |
| Test | 33 | 27 | 60 |

Table S1 The composition of the dataset

Table S2 Performance on the test set

| Metric | Test performance |
| --- | --- |
| Accuracy | 81.67% |
| Precision | 100% |
| Recall (sensitivity) | 66.67% |
| F1-score | 80.00% |

Table S3 The corresponding confusion matrix of the test set

|  | Predicted 10 μL | Predicted >10 μL |
| --- | --- | --- |
| Actual 10 μL | 22 | 11 |
| Actual >10 μL | 0 | 27 |
